# Monitoring or Colluding? Institutional Investors' Heterogeneity and Environmental Information Disclosure Behavior

**DOI:** 10.3389/fpsyg.2022.911901

**Published:** 2022-06-16

**Authors:** Zhengguang Li, Tinghai Zhang, Xibo Zhao, Yicheng Zhu

**Affiliations:** ^1^School of Economics and Management, Yancheng Institute of Technology, Yancheng, China; ^2^Center for Economic Development Research, Anhui University of Finance and Economics, Bengbu, China; ^3^School of Business, Renmin University of China, Beijing, China

**Keywords:** environmental information disclosure behavior, environmental information disclosure quality, institutional investors' heterogeneity, China, the stable institutional investors, the unstable institutional investors

## Abstract

High-quality environmental information disclosure is not only an effective way for the firm to fulfill its environmental responsibility and promote green development, but also an important governance mechanism to reduce the degree of information asymmetry between the firm's management and shareholders and alleviate the agency conflict. As an important shareholder of a firm, there are two different hypotheses about the influence of institutional investors on firm decision-making and behavior: monitor and collusion. Institutional investors are not homogeneous, and there are significant differences in the impact of different types of institutional investors on firm decision-making and behavior. We divide institutional investors into the stable institutional investors and the unstable institutional investors, using the data of listed firms in China's A-share heavy pollution industry between 2008 and 2020, and this study explores the effect of institutional investors' heterogeneity on environmental information disclosure behavior from the perspective of environmental information disclosure quality. Empirical evidence shows that institutional investors as a whole have a positively significant impact on environmental information disclosure quality. Further analysis shows that the stable institutional investors have positive impact on environmental information disclosure quality compared with the unstable institutional investors. After a series of robustness tests, the conclusion is still valid. The results of this paper show that institutional investors, especially the stable institutional investors, can effectively reduce the degree of information asymmetry, alleviate the agency conflict of the firm, play an active role in corporate governance, strengthen the main responsibility of firm ecological environment protection, and promote the green development of firm. The conclusion of this paper has important reference significance for the regulators to formulate policies to improve environmental information disclosure quality and promote green development according to the heterogeneity of institutional investors.

## Introduction

As the main participants in the market, whether firms can fulfill their environmental responsibilities, strengthen environmental protection, and control environmental pollution plays an important role in promoting green development. To strengthen firms to fulfill their environmental responsibilities, the Chinese government has strengthened the supervision of firms' environmental information disclosure behavior, hoping to promote the green and low-carbon development of firms through the effective tool of environmental information disclosure. Ministry of Ecology and Environment of the People's Republic of China promulgated the administrative measures for the legal disclosure of firm environmental information in December 2021, which defines the basic contents of the legal disclosure of firm environmental information, such subject, form, time limit, supervision, and management, strengthens the entity responsibility of firm ecological and environment protection, and standardizes environmental information disclosure activities in accordance with the law. High-quality environmental information disclosure is not only an important embodiment of firm environmental information disclosure behavior, but also an important governance mechanism to reduce information asymmetry between the firm's management and shareholders and alleviate the agency conflict. Therefore, it is of important theoretical and practical significance to investigate the impact factors of environmental information disclosure behavior from the perspective of environmental information disclosure quality.

In the recent years, the number and shareholding ratio of institutional investors in China have gradually increased, and institutional investors have become an important shareholder of the firm. There are two different hypotheses of monitor and collusion in the impact on the firm's behavior and decision-making. Institutional investors are not homogeneous, and there are significant differences in the impact of different types of institutional investors on firm behavior and decision-making. So, what is the impact of institutional investors on firm environmental information disclosure? Is there any difference in the impact of different institutional investors on the firm's environmental information disclosure? This is an important proposition to be tested. Existing literature mainly examines the impact of institutional investors on firm behavior and decision-making from the perspectives of dividend policy (Grinstein and Michaely, 2005), earning management (Chung et al., 2002; Hsu and Koh, 2005; Wang, 2014), and capital structure (Chung and Wang, 2014). Few literature examines the corporate governance effect of institutional investors from the perspective of environmental information disclosure behavior. Therefore, based on the method of Elyasiani and Jia (2010), this paper divides institutional investors into the unstable and the stable institutional investors, examines the impact of institutional investors on environmental information disclosure behavior from the perspective of environmental information disclosure quality, and hopes to test whether there are differences in the corporate governance effects of heterogeneous institutional shareholders from the perspective of environmental information disclosure quality.

To solve the above problems, based on the dividing institutional investors into stable and unstable institutional investors, this paper uses the data of listed firms in China's A-share heavy pollution industry between 2008 and 2020 and examines the impact of institutional investor heterogeneity on environmental information disclosure behavior from the perspective of environmental information disclosure quality. The reasons for choosing the heavy pollution industry as the research sample are as follows: On the one hand, compared with the light pollution industry, the heavy pollution industry, as the main manufacturer of environmental pollution, has a more serious impact on the human living environment; on the other hand, some laws issued by China require heavy pollution industry to disclose environmental information mandatorily, whereas only light pollution industries are required to disclose environmental information voluntarily. It can be seen that the mandatory provisions on environmental information disclosure in the heavy pollution industry ensure that they disclose more sufficient and comprehensive environmental data. Therefore, this paper takes the listed companies in the heavy pollution industry as the research sample.

Empirical evidence shows that the higher the shareholding ratio of institutional investors, the higher environmental information disclosure quality, thus verifying the effective monitor hypothesis of institutional investors' impact on firm environmental information disclosure behavior. Further analysis indicates that compared with the unstable institutional investors, the stable institutional investors have significant positive impact on environmental information disclosure quality. After a series of robustness tests, the research conclusion is still valid. The results of this paper show that institutional investors, especially the stable institutional investors, can effectively monitor the environmental information disclosure behavior of firms, strengthen the main responsibility of firm ecological and environmental protection, and promote the green development of firms. The research conclusion of this paper has important reference significance for the regulators to formulate policies to improve environmental information disclosure quality and promote green development according to the institutional investors' heterogeneity.

This paper has the following contributions: First, it enriches the literature in the field of impact factors of environmental information disclosure quality. Existing literature mainly examines the impact factors of environmental information disclosure quality from the perspectives of government regulation (Darrell and Schwartz, 1997; Cho and Patten, 2007; Huang and Kung, 2010), corporate governance (Brammer and Pavelin, 2006; Zeng et al., 2012), firm characteristics (Cormier et al., 2005; Brammer and Pavelin, 2008; Lu and Abeysekera, 2014; Ismail et al., 2018), executive characteristics (Lewis et al., 2014), culture and institution (Buhr and Freedman, 2001), and media attention (Rupley et al., 2012; Solikhah and Maulina, 2021). This paper examines the impact factors of environmental information disclosure quality from the perspective of institutional investors, thus broadening the research boundary of impact factors of environmental information disclosure quality. Second, it expands the research on the field of economic consequences of institutional investors. Existing literature mainly investigates economic consequences of institutional investors from the perspectives of dividend policy (Grinstein and Michaely, 2005), earning management (Chung et al., 2002; Hsu and Koh, 2005; Wang, 2014), and capital structure (Chung and Wang, 2014). Taking environmental information disclosure quality as the starting point, this paper examines the economic consequences of institutional investor heterogeneity, thus deepening the research of institutional investors' impact on firm behavior and decision-making. Finally, the research conclusions of this paper are helpful to provide reference for regulators to manage environmental information disclosure according to institutional investors' heterogeneity. This paper reveals that compared with the unstable institutional investors, the stable institutional investors have a significant positive impact on environmental information disclosure quality. The conclusion of this paper provides a reference for regulators to fully consider institutional investors' heterogeneity in the process of environmental information disclosure management.

The remainder of this paper is structured as follows: Literature and Research Hypotheses reviews the literature and develops the hypothesis. Research Design introduces the research design. Empirical Results presents the empirical results. ROBUSTNESS TESTS is the robustness test. Additional Analysis makes additional analysis. Finally, Conclusions concludes the paper.

## Literature and Research Hypotheses

Since this paper examines the impact of institutional investors' heterogeneity on firm environmental information disclosure behavior from the perspective of environmental information disclosure quality, this paper only reviews the literature in three fields of impact factors of environmental information disclosure quality, corporate governance effect of institutional investors, and types of institutional investors.

### Impact Factors of Environmental Information Disclosure Quality

Environmental information disclosure is an effective environmental protection management tool. High-quality environmental information disclosure is not only conducive to the government to understand the performance of firm environmental responsibilities, but also conducive to investors to measure the attitude of firm to bear social responsibility and the level of environmental governance, enhance their confidence in firm management, and promote the green development of firm. So, what factors will affect environmental information disclosure quality? This has become the hot topic of academics. A large number of literature mainly investigate the impact factors of environmental information disclosure quality from the perspectives of government regulation, corporate governance, corporate characteristics, executive characteristics, culture and institution, and media attention. For example, in terms of government regulation, Darrell and Schwartz (1997), Cho and Patten (2007), and Huang and Kung (2010) find that the regulatory requirements of the government and the pressure of public policy significantly affect the environmental information disclosure behavior of firm. In terms of corporate governance, Brammer and Pavelin (2006) investigate the impact of ownership structure on environmental information disclosure and find that the more decentralized the ownership structure of a company, the more willing it is to make voluntary information disclosure. Zeng et al. (2012) use China's A-share listed firms from 2006 to 2008 as the research sample. The research shows that the greater the separation of corporate control and cash flow rights, the less likely it is to disclose more environmental information. In terms of organizational impression and reputation, Zeng et al. (2012) find that firms with “China famous brand” trademark disclose more environment information than other firms. In terms of firm characteristics, Cormier et al. (2005), Brammer and Pavelin (2008), Lu and Abeysekera (2014), and Ismail et al. (2018) find that firm size, profitability, financial leverage, risk, and fixed assets are the influencing factors of environmental information disclosure quality. In terms of executive characteristics, Lewis et al. (2014) investigate the impact of executive tenure and educational background on environmental information disclosure quality. The study finds that newly appointed CEOs and CEOs with MBA degrees are more likely to disclose environment information, whereas CEOs with legal background are less likely to disclose environmental information. In terms of culture and institution, Buhr and Freedman (2001) discuss the impact of cultural and institutional factors on environmental information disclosure by comparing firms in Canada with the United States. The study finds that Canadian companies disclosed more environmental information than American companies. This shows that the Canadian Environment and culture basis of information disclosure is more conducive than that of the United States. They also point out that Canada, as a collectivist society, leads to a greater degree of voluntary environmental information disclosure, especially in environmental reports. However, as a society with high litigation rate, the United States has more mandatory disclosures in the annual report. In terms of media attention, Rupley et al. (2012) and Solikhah and Maulina (2021) find that the more media reports, the higher environmental information disclosure quality.

### Corporate Governance Effect of Institutional Investors

Institutional investors have developed rapidly at home and abroad, and their shareholding ratio has been significantly increased. Institutional investors have become the major shareholders of the capital market. Can institutional investors play the role of corporate governance as government regulators expected? At present, there are two views on the corporate governance effect of institutional investors:

One view is that institutional investors cannot exert a positive corporate governance effect. Bushee and Noe (2000) find that high-quality information disclosure attracted the shareholding of short-term institutional investors, and the increase in the shareholding proportion of short-term institutional investors exacerbated the volatility of the firm's stock return. Graves and Waddock (1994) believe that the increase in shareholding ratio of institutional investors leads to a decline in corporate competitiveness and financial performance. Since the returns of institutional investors are determined on a quarterly basis (Graves and Waddock, 1994), they pursue short-term returns. Therefore, this view attributes a part of the decline in corporate competitiveness and financial performance to the need for fund managers to frequently prove that their investment returns are constantly improving. In response to institutional investors' pursuit of short-term returns, the firm's management does not consider managing the firm from the perspective of long-term interests, resulting in a decline in firm performance and competitiveness (Johnson and Greening, 1999).

However, another view is that with the significant increase in the shareholding ratio of institutional investors, institutional investors can play a positive monitor role compared with investors with small shareholding ratio, negative and closed information. Brickley et al. (1988), Holderness and Sheehan (1988), Gilson and Kraakman (1991), Pound (1992), Hoskisson et al. (1994), Almazan et al. (2005), Aggarwal et al. (2011), Chung and Zhang (2011), and others believe that due to the high shareholding of institutional investors, the purchase and sale of a firms' shares will have a significant impact on the stock price of the firm. Their results show that the institutional investors are more interested in not only their financial performance, but also the strategy and business activities of their investment firm than other shareholders of the firm. They are more likely to participate in the decision and further improve corporate governance. Ferreira and Matos (2008) find that firms with higher shareholding of institutional investors have less capital expenditure, indicating that institutional investors reduce over-investment. Parrino et al. (2003) and Aggarwal et al. (2011) find that institutional investors can force CEO change through positive monitor behavior. They believe that institutional investors can directly affect decision of the board to achieve the purpose of replacing CEO. If the firm with poor operating performance did not change CEO, then institutional investors can also use the indirect way of selling its shares to impact the firm. Hartzell and Starks (2003) find that the shareholding of institutional investors positively correlates with executive performance pay sensitivity and negatively correlates with executive pay level, which provides direct evidence that institutional investors affect the compensation structure of corporate executives. Chen et al. (2008), Ruiz-Mallorquí and Santana-Martin (2011), Abukosim et al. (2014), Thanatawee (2014), and Guo and Platikanov (2019) find that the higher the shareholding ratio of institutional investors, the greater the firm value. Abbasi et al. (2012) and Petta and Tarigan (2017) find that the higher the shareholding ratio of institutional investors, the higher the efficiency of the firm's management. According to Dang et al. (2018), the shareholding ratio of institutional investors positively correlates with stock liquidity.

Due to the existing literature about institutional investors' corporate governance effect has two completely different views, many scholars try to seek theoretically the reason of the two different governance effects, which is more representative. Pound (1992) proposes three hypotheses about whether institutional investors can play a role in the process of corporate governance: effective monitor hypothesis, ineffective monitor hypothesis, and interest collusion hypothesis. The effective monitor hypothesis means that institutional investors can make use of the information, professional, and talent advantages of their major shareholders to effectively monitor firm management. This effective monitor can increase the value of the firm, and institutional investors can gain benefits over the monitor cost from this monitor. If the institutional investors are not satisfied with the operating performance or decision of board of the investment firm, they can put pressure on the management of the firm by selling their shares and adopting active strategies. The ineffective monitor hypothesis means that institutional investors take trading as their main purpose, have short-sighted behavior, do not interfere with corporate governance, and decide to hold or sell stocks according to the balance of their portfolio. The interest collusion hypothesis refers to the collusion between institutional investors and management to occupy the interests of dispersed minority shareholders. For example, to obtain more investment banking business, investment firms often support the management at the expense of minority shareholders.

### Institutional Investors and Information Disclosure

Existing literature shows that institutional investors' shareholding can improve the level of information disclosure. For example, Boone and White (2015) find that the higher the shareholding ratio of institutional investors, the fuller the management disclosure and the more analysts follow-up, thus reducing information asymmetry. Ilhan et al. (2021) find that institutional investors attach importance to and need climate risk disclosure information. It can be seen that although academia has investigated the impact of institutional investor shareholding on information disclosure from the perspective of climate risk information disclosure, these research conclusions are based on the data of western developed countries. The system of western countries is different from that of China. As an emerging transition economy, can institutional investor shareholding play a supervisory role, promote listed firms to fulfill their environmental responsibility, and improve environmental information disclosure quality? This is a research motivation of this paper.

### Types of Institutional Investors

Many studies indicate that not all institutional investors are the same (Brickley et al., 1988; Bushee, 1998; Almazan et al., 2005; Chen et al., 2007; Elyasiani and Jia, 2010; Bushee et al., 2014). Different types of institutional investors have different initiatives to monitor the firm's management. At present, the criteria for the types of institutional investors mainly include the following six types:

First, Brickley et al. (1988) divide institutional investors into pressure-non-sensitive institutional investors and pressure-sensitive institutional investors according to whether institutional investors have an existing or potential commercial relationship with the invested firm. Second, Bushee (1998) divides institutional investors into short-term institutional investors, long-term institutional investors, and quasi-index institutional investors according to the expected investment period of institutional investors. Third, Almazan et al. (2005) believe that institutional investors play an important role in monitoring the behavior of firm management, but not all institutional investors can play the same role. They divide institutional investors into two types based on different monitor costs: potential positive institutional investors and potential negative institutional investors. Fourth, Chen et al. (2007) showed that in the context of cost-benefit, institutional investors are divided into monitor and short-term institutional investors. Fifth, Bushee et al. (2014) showed according to the preference of institutional investors for the improvement of their investment corporate governance mechanism, institutional investors are divided into sensitive institutional investors and insensitive corporate governance institutional investors. Sixth, Elyasiani and Jia (2010) divides institutional investors into the stable institutional investors and the unstable institutional investors according to the difference of investment horizon and shareholding motivation. The stable institutional investors refer to the institutional investors who pay attention to their investment firms for a long time and can actively participate in corporate governance and monitor the behavior of firm management. They are long-line investors of listed firms. The unstable institutional investors have obvious speculative shareholding in listed firms and always want to gain benefits according to the fluctuation of stock prices. They are short-term investors of listed firms. These two types of institutional investors have different enthusiasm to participate in corporate governance.

This paper believes that Elyasiani and Jia (2010) is a more reasonable type of institutional investors. Therefore, when examine the impact of institutional investors on environmental information disclosure quality, we divide institutional investors into the unstable and the stable institutional investors.

### Research Hypotheses

#### Institutional Investors and Environmental Information Disclosure Quality

The basic feature of modern firm system is the separation of ownership and management. One problem brought by the separation of the two rights is the agency conflict and information asymmetry between shareholders and management (Jensen and Meckling, 1976). In the case of information asymmetry, management know more about the production and operation activities of the firm than the shareholders and have private information about the production and operation activities of the firm. When management's personal objective function and the shareholder objective function are inconsistent, management may for personal interests (e.g., empire building), only focus on the firm's short-term earnings, and implement behavior that may harm the interests of shareholders, and make management activities have short-sighted behavior. These agency problems will damage the value of the firm.

According to the theory of corporate governance, the governance subject of modern firms has experienced a process of expanding the scope and extending the boundary. The governance subject of modern firms has expanded from the past shareholders to all firm members including shareholders, creditors, operators, and employees and then to a wider range of stakeholders. Stakeholders have a strong motivation to participate in corporate governance, but in an emerging transition country such as China, the imperfect development of capital market, unreasonable shareholding structure, and insufficient information disclosure lead to the inability of some stakeholders to monitor management. Institutional investors are endowed by the country to improve the governance of listed firms because of their industry expertise, information advantages, and capital advantages, as well as their high shareholding ratio in investment firms (Shleifer and Vishny, 1986; Huddart, 1993; Karpoff et al., 1996; Maug, 1998; Gillan and Starks, 2000).

Academics generally believe the environmental information disclosure, as a corporate governance mechanism (Criado-Jimenez et al., 2008; Evans et al., 2009; Zeng et al., 2012), high-quality environmental information disclosure can reduce information asymmetry between management and external stakeholders and alleviate agency conflict, which will also indirectly lead to the increase in firm value and stock value. Therefore, theoretically, shareholders need high-quality environmental information disclosure to reduce the degree of information asymmetry, alleviate agency conflict, and protect their own interests from infringement. However, not all shareholders of the firm need high-quality environmental information disclosure. Only large shareholders with information advantages, professional advantages, and financial advantages such as institutional investors can actively supervise the daily information disclosure of the firm's management when the benefit obtained from the supervision of their investment company is higher than the cost.

With the implementation of the policy of vigorously developing institutional investors issued by the Chinese government in the recent years, institutional investors have become an important shareholder of Chinese listed firms (Khan et al., 2005) and have a stronger ability to supervise the firm's management. If high-quality environmental information disclosure can provide governance benefits, institutional investors are more likely to understand and evaluate such governance benefits. Therefore, from this perspective, institutional investors need to obtain high-quality environmental information disclosure reports from firm management.

Based on the above analysis, we propose hypothesis 1 of this paper:

H1: Ceteris paribus, shareholding ratio of institutional investors positively correlates with environmental information disclosure quality.

#### Institutional Investors' Heterogeneity and Environmental Information Disclosure Quality

Previous studies show that institutional investors are not homogeneous, and there are significant differences in the monitor of the daily activities of the management of the invested firms (Brickley et al., 1988; Bushee, 1998; Almazan et al., 2005; Chen et al., 2007; Elyasiani and Jia, 2010; Bushee et al., 2014). This paper also follows this view, drawing on the research of Elyasiani and Jia (2010) and dividing institutional investors into the stable institutional investors and the unstable institutional investors according to their different investment horizon and shareholding motivation. The characteristics of the stable institutional investors are that they pay attention to the listed firms they invest in for a long time, actively participate in corporate governance, and monitor the behavior of management. They are long-term investors of listed firms. The characteristics of the unstable institutional investors are that the shareholding of listed firms invested by them is obviously speculative. They do not pay attention to the long-term operation and profitability of listed firms and always hope to gain benefits through short-term stock price volatility.

Since high-quality environmental information disclosure is based on the firms' full performance of environmental protection responsibilities and increasing environmental protection investment, it requires expensive expenditure, which will produce benefits in a long period of time and will lead to the decline of firm profits in the short term. For the unstable institutional investors, they hope to obtain benefits through short-term stock price volatility, but high-quality environmental information disclosure will lead to sharp volatility in the firm's stock price (Bushee and Noe, 2000), which will affect the interests of unstable institutional investors. Therefore, the unstable institutional investors have no motivation to supervise the performance of the firm management's environmental responsibility, so environmental information disclosure quality will not be improved. High-quality environmental information disclosure is not only conducive to investors' decision-making, but also to correct the market pricing related to environmental information. Therefore, compared with the unstable institutional investors, the stable institutional investors need high-quality environmental information disclosure to reduce information asymmetry and alleviate agency conflict.

Based on the above analysis, we propose hypothesis 2 of this paper:

H2: Ceteris paribus, compared with the unstable institutional investors, the stable institutional investors have positive impact on environmental information disclosure quality.

## Research Design

### Sample Selection and Data Source

This study uses the listed firms in China's A-share heavy pollution industry between 2008 and 2020 as the research sample and selects the listed firms with Chinese A-share industry codes of B, C, and D as the heavy pollution firms according to the classified management directory of environmental protection verification industry of China's listed firms and the industry classification guidelines of China's listed firms (revised in 2012). The reasons for choosing the heavy pollution industry as the research sample are as follows: On the one hand, compared with the light pollution industry, the heavy pollution industry, as the main manufacturer of environmental pollution, has a more serious impact on the human living environment; on the other hand, some laws issued by China require heavy pollution industry to disclose environmental information mandatorily, whereas only light pollution industries are required to disclose environmental information voluntarily. It can be seen that the mandatory provisions on environmental information disclosure in the heavy pollution industry ensure that they disclose more sufficient and comprehensive environmental data. Therefore, this paper takes the listed companies in the heavy pollution industry as the research sample.

The sample screening process is as follows: First, we excluded firms in special processing status (ST, ^*^ST) during the sample period; Second, we exclude the observation value with missing data; Finally, to eliminate the influence of extreme values, we winsorize all continuous variables at the 1 and 99 percentiles. Through the above sample screening process, a total of 14,358 firm-year observations are obtained in this paper. The financial data and environmental information disclosure quality data required in this paper are from China Securities Market and Accounting Research database (CSMAR database).

### Measuring Environmental Information Disclosure Quality

This study evaluates 25 specific disclosure items in five categories, including environmental management disclosure, environmental certification disclosure, environmental information disclosure carrier, environmental liability disclosure, and environmental performance and governance disclosure, reported in the environmental research sub-database contained in China Securities Market and Accounting Research database (CSMAR database). On this basis, the scores of each disclosure item are aggregated, and the natural logarithm is taken as the proxy variable of environmental information disclosure quality (*Eidq*). The larger the indicator, the higher environmental information disclosure quality.

Based on the ideas of Wiseman (1982), the value is assigned according to whether to disclose environmental information of the firm with monetary information: (1) for the items disclosed with monetary information, the value of quantitative and qualitative disclosure is 2, the value of qualitative disclosure is 1, and the value of non-disclosure is 0. (2) For the items with non-monetary information disclosure, the value of disclosure is 2, and the value of non-disclosure is 0. Specifically, the items in environmental liability disclosure, environmental performance, and governance disclosure belong to monetary information items; the items in environmental management disclosure carriers, environmental certification disclosure, and environmental information disclosure belong to non-monetary information items. The specific scoring criteria for environmental information disclosure items are shown in Table 1.

**Table 1:** Scoring criteria for environmental information disclosure items.

**Disclosure type**	**Disclosure items**	**Score instructions**
Environmental management disclosure	Environmental protection concept	Disclosure: 2 points No:0 points
	Environmental protection objectives	
	Environmental protection management system	
	Environmental protection education and training	
	Special action on environmental protection	
	Environmental time emergency mechanism	
	Environmental protection honors or awards	
	The “Three Simultaneous” system	
Environmental certification disclosure	Whether it has passed ISO14001 certification	Yes: 2 points No:0 points
	Whether it has passed ISO9001 certification	
Environmental information disclosure carrier	Annual report of listed companies	Disclosure: 2 points No:0 points
	Social responsibility report	
	Environmental report	
Disclosure of environmental liabilities	Wastewater discharge	Quantitative and qualitative description: 2 points Only qualitative: 1 point No:0 pois
	COD emissions	
	SO_2_ emissions	
	CO_2_ emissions	
	Smoke and dust emissions	
	Industrial solid waste emissions	
Environmental performance and Governance disclosure	Waste gas emission reduction and treatment	
	Wastewater emission reduction and treatment	
	Dust and smoke treatment	
	Utilization and disposal of solid waste	
	Control of noise, light pollution, radiation, etc.	
	Implementation of cleaner production	

### Measurement of Institutional Investors' Heterogeneity

Drawing on the research of Elyasiani and Jia (2010), this paper divides institutional investors into the unstable and the stable institutional investors from dimensions of horizon and industry. The specific calculation formula is as follows:


(1) $$\{\begin{array}{l} Sd_{it}=\frac{Invh_{it}}{Std(Invh_{it-3},Invh_{it-2},Invh_{it-1})}\\ Stable_{it}=\{\begin{array}{l} 1,Sd_{it}\geq Median_{tj}(Sd_{tj})\\ 0,Otherwise \end{array} \end{array}$$

In model (1), *Invh*_*it*_ represents the shareholding ratio of institutional investors of firm *i* in year *t*; *Std*(*Invh*_*it*−3_, *Invh*_*it*−2_, *Invh*_*it*−1_)represents the standard deviation of the shareholding ratio of institutional investors previous 3 years of the firm *i*; *Sd*_*it*_ represents the institutional investor heterogeneity from the horizon dimension; *Median*_*tj*_(*Sd*_*tj*_) represents the median of industry *j* in year *t*; *Stable*_*it*_ is a dummy variable, which means measuring the institutional investors heterogeneity from the industry dimension. When *Sd*_*it*_≥*Median*_*tj*_(*Sd*_*tj*_), the value is 1, it means that the institutional investor of firm *i* in year *t is a* stable institutional investor; otherwise, the value is 0, it means that the institutional investor of firm *i* in year *t* is a unstable institutional investor.

### Model Specification

#### Model Specification for Hypothesis 1

Based on the research of Zeng et al. (2012), Lu and Abeysekera (2014), Ismail et al. (2018), and Fan et al. (2020), the following model (2) is used to test hypothesis 1:


(2) $$\begin{array}{l} Eidq=\beta_{0}+\beta_{1}Invh+\beta_{2}Roa+\beta_{3}Growth+\beta_{4}Lev\\ +\beta_{5}Size+\beta_{6}Board\\ +\beta_{7}Ddbl+\beta_{8}Dual+\sum^{\text{​}}Year+\sum^{\text{​}}Industry+\varepsilon \end{array}$$

In model (1), *Ediq* represents environment information disclosure quality, which is measured by the methods listed in Measurement of Institutional Investors' Heterogeneity; *Invh* represents the shareholding ratio of institutional investors, which is measured by dividing the number of common stock held by institutional investors by the total number of common stock; *Roa* represents firm profitability, which is measured by dividing the net profit by the average total assets; *Growth* represents firm growth, which is measured by the growth rate of sales; *Lev* represents firm leverage, which is measured by dividing the year-end total liabilities by the total assets; *Size* represents firm size, which equals to the natural logarithm of total assets; *Board* represents the size of the board of directors, which equals to the natural logarithm of the number of directors; *Ddbl* represents the proportion of independent directors, which is measured by dividing the number of independent directors by the number of the board of directors; *Dual* is a dummy variable that equals 1 if the chairman is also the CEO and 0 otherwise; *Year* represents the year dummy variable; *Industry* represents the industry dummy variable; ε is a random error term.

#### Model Specification for Hypothesis 2

Based on method of Zeng et al. (2012), Lu and Abeysekera (2014), Ismail et al. (2018), and Fan et al. (2020), the following model (3) is used to test hypothesis 2:


(3) $$\begin{array}{l} Eidq=\beta_{0}+\beta_{1}Stable+\beta_{2}Roa+\beta_{3}Growth\\ +\beta_{4}Lev+\beta_{5}Size+\beta_{6}Board\\ +\beta_{7}Ddbl+\beta_{8}Dual+\sum^{\text{​}}Year+\sum^{\text{​}}Industry+\varepsilon \end{array}$$

In the model (3), *Stable* represents institutional investors heterogeneity, which is calculated by formula (1). If the value is 1, it means that the institutional investors of the firm are stable institutional investors, otherwise it is 0, which indicate that the firm's institutional investors are unstable institutional investors. The definition of other variables is the same as that of model (2). Specific definitions of the variables are detailed in Table 2.

**Table 2:** Definition of relevant variable.

**Variable**	**Variable code**	**Variable definition**
Environmental information disclosure quality	*Ediq*	The natural logarithm is taken after the sum of Table 1 assignment method
Shareholding ratio of institutional investors	*Invh*	The number of common stocks held by institutional investors divided by the total number of common stocks
Institutional investors heterogeneity	*Stable*	Calculated by formula (1)
Firm profitability	*Roa*	Net profit divided by the average total assets
Firm growth	*Growth*	Growth rate of the firm's operating revenue
Financial leverage	*Lev*	Total liabilities at the end of the year divided by total assets at the end of the year
Firm size	*Size*	Natural logarithm of total assets at the end of the year
Board size	*Board*	Natural logarithm of the number of directors of the firm at the end of the year
Proportion of independent directors	*Ddbl*	The number of independent directors at the end of the year divided by the number of directors at the end of the year
Duality	*Dual*	Dummy variable, if the chairman is also the general manager, the value is 1; Otherwise, the value is 0

## Empirical Results

### Descriptive Statistics

Table 3 reports the descriptive statistical results of the main variables. From Table 3, mean (median) of environmental information disclosure quality (*Eidq)* is 2.039 (2.079), and standard deviation is 0.913, which indicates that there is no significant difference in environment information disclosure quality (*Eidq)* within the sample. Mean (median) of shareholding ratio of institutional investors (*Invh)* is 41.70% (43.80%), and the standard deviation is 0.246, which indicates that there is no significant difference in shareholding ratio of institutional investors (*Invh)* within the sample. Mean of institutional investor heterogeneity (*Stable)* is 0.513, which indicates that the stable institutional investors of listed firms in China account for the majority.

**Table 3:** Descriptive statistics of the main variables.

**Variable**	**Observations**	**Mean**	**Std Dev**	**Min**	**Q1**	**Median**	**Q3**	**Max**
*Eidq*	14,358	2.039	0.913	0	1.386	2.079	2.773	3.892
*Invh*	14,358	0.417	0.246	0.001	0.206	0.438	0.610	0.902
*Stable*	14,358	0.513	0.500	0	0	1	1	1
*Roa*	14,358	0.051	0.066	−0.243	0.026	0.049	0.082	0.238
*Growth*	14,358	0.167	0.420	−0.497	−0.024	0.100	0.252	3.043
*Lev*	14,358	0.428	0.197	0.059	0.272	0.425	0.576	0.900
*Size*	14,358	22.259	1.244	19.976	21.376	22.082	22.958	26.037
*Board*	14,358	2.140	0.202	1.099	1.946	2.197	2.197	2.890
*Ddbl*	14,358	0.374	0.053	0.333	0.333	0.333	0.429	0.571
*Dual*	14,358	0.260	0.439	0	0	0	1	1

### Correlation Analysis

The Pearson (spearman) correlation coefficient between related variables is reported in Table 4. We can find that the Pearson (spearman) correlation coefficient between the shareholding ratio of institutional investors and environmental information disclosure quality is significantly positive, which indicates that univariate analysis has verified H1. The Pearson (Spearman) correlation coefficient between institutional investors' heterogeneity and environmental information disclosure quality is significantly positive, which indicates that the univariate analysis has verified H2. The correlation coefficients between other variables are below 0.6, indicating that there is no serious multicollinearity. To control the impact of other factors on environmental information disclosure quality, we conduct multiple regression analysis below.

**Table 4:** Pearson (Spearman) correlation coefficient.

**Variable**	***Eidq***	***Invh***	***Stable***	***Roa***	***Growth***	***Lev***	***Size***	***Board***	***Ddbl***	***Dual***
*Eidq*	1	0.215***	0.148***	0.087***	−0.043***	0.150***	0.393***	0.184***	−0.037***	−0.110***
*Invh*	0.210***	1	0.517***	0.127***	−0.005	0.193***	0.407***	0.216***	−0.071***	−0.179***
*Stable*	0.145***	0.522***	1	0.008	−0.086***	0.094***	0.163***	0.121***	−0.055***	−0.116***
*Roa*	0.094***	0.132***	0.028***	1	0.327***	−0.248***	0.087***	0.035***	−0.036***	0.016*
*Growth*	−0.057***	0.020**	−0.100***	0.226***	1	−0.011	0.050***	−0.023***	0.003	0.049***
*Lev*	0.139***	0.194***	0.097***	−0.278***	0.022***	1	0.487***	0.179***	−0.030***	−0.119***
*Size*	0.402***	0.425***	0.175***	0.097***	0.049***	0.467***	1	0.258***	−0.031***	−0.160***
*Board*	0.189***	0.227***	0.124***	0.053***	−0.022***	0.183***	0.291***	1	−0.552***	−0.200***
*Ddbl*	−0.038***	−0.075***	−0.048***	−0.034***	−0.004	−0.030***	−0.018**	−0.514***	1	0.105***
*Dual*	−0.108***	−0.186***	−0.116***	0.001	0.027***	−0.119***	−0.148***	−0.190***	0.107***	1

The lower left corner (upper right corner) is Pearson (Spearman) correlation coefficient, *, **, ***, respectively, indicate significant at the level of 10, 5, and 1%.

### Multiple Regression Results

Table 5 reports the multiple regression results for the impact of institutional investors' heterogeneity on environmental information disclosure quality. Column (1) of Table 5 reports the multiple regression results of the impact of shareholding ratio of institutional investors on the environmental information disclosure quality; column (2) of Table 5 reports the multiple regression results of the impact of institutional investors heterogeneity on environmental information disclosure quality.

**Table 5:** Institutional investors' heterogeneity and environmental information disclosure quality.

**Variable**	**(1)**	**(2)**
*Intercept*	−4.726*** (−25.14)	−4.807*** (−26.09)
*Invh*	0.153*** (4.90)	
*Stable*		0.104*** (7.49)
*Roa*	0.936*** (8.07)	0.950*** (8.23)
*Growth*	−0.172*** (−10.19)	−0.159*** (−9.39)
*Lev*	−0.085** (−1.96)	−0.094** (−2.17)
*Size*	0.258*** (35.33)	0.264*** (37.75)
*Board*	0.448*** (10.49)	0.443*** (10.38)
*Ddbl*	0.317** (2.09)	0.310** (2.04)
*Dual*	−0.093*** (−5.84)	−0.092*** (−5.81)
*Year fixed effect*	Yes	Yes
*Industry fixed effect*	Yes	Yes
*Observations*	14,358	14,358
*Adj. R2*	0.212	0.214

t-statistics are in parentheses; ** and *** indicate significance at the 5 and 1% level, respectively.

Column (1) of Table 5 shows that there is a positive correlation between *Invh* and *Eidq* (β_1_=0.153, *t*=4.90), which is significant at the level of 1%, and shows that the higher the shareholding ratio of institutional investors, the higher firm environmental information disclosure quality. That is, institutional investors play an effective role in monitoring the firm's environmental information disclosure behavior, monitor firms to fulfill environmental responsibilities, fully disclose environmental information, and improve environmental information disclosure quality, so that H1 of this paper is verified.

In terms of control variables, there is a positive correlation between *Roa* and *Eidq* (β_2_ = 0.936, *t* = 8.07), which is significant at the level of 1%, indicating that the stronger the profitability, the higher environmental information disclosure quality, which is consistent with the research conclusions of Lu and Abeysekera (2014) and Ismail et al. (2018). There is a negative correlation between the *Growth* and *Eidq* (β_3_= −0.172, *t* = −10.19), which is significant at the level of 1%, which indicates that the better the growth of firm, the worse environmental information disclosure quality, and consistent with the research conclusion of Fan et al. (2020). There is a negative correlation between *Lev* and *Eidq* (β_4_ = −0.085, *t* = −1.96), which is significant at the level of 5%, indicating that the higher the financial leverage, the worse environmental information disclosure quality. It is consistent with the research conclusions of Ismail et al. (2018) and Fan et al. (2020). There is a positive correlation between *Size* and *Eidq (*β_5_ = 0.258, *t* = 35.33), which is significant at the level of 1%, indicating that the larger the firm size, the higher environmental information disclosure quality. It is consistent with the research conclusions of Zeng et al. (2012), Lu and Abeysekera (2014), Ismail et al. (2018), and Fan et al. (2020). There is a positive correlation between *Board* and *Eidq* (β_6_ = 0.448, *t* = 10.49), and it is significant at the level of 1%, which indicates that the larger the size of the board of directors, the higher environmental information disclosure quality. There is a positive correlation between *Ddbl* and *Eidq* (β_7_ = 0.317, *t* = 2.09), and it is significant at the level of 5%, which indicates that the higher the proportion of independent directors, the higher environmental information disclosure quality. There is a negative correlation between *Dual* and *Eidq* (β_8_ = −0.093, *t* = −5.84), and it is significant at the level of 1%, which indicates that environmental information disclosure quality is worse in the firm where CEO duality.

Column (2) of Table 5 reveals that there is a positive correlation between *Stable* and *Eidq* (β_1_ = 0.104, *t* = 7.49), and it is significant at the level of 1%, which indicates that compared with the unstable institutional investors, the stable institutional investors have positive impact on environmental information disclosure quality. Therefore, compared with the unstable institutional investors, the stable institutional investors are effective monitor. They effectively monitor the firm's environmental information disclosure behavior, promote the company to fully disclose environmental information, and improve environmental information disclosure quality. Thus, the H2 of this paper is verified.

In terms of control variables, the research conclusions in column (2) of Table 5 are consistent with those in column (1) of Table 5.

## Robustness Tests

### Endogenous Tests

So far, our research conclusions show that the higher the shareholding ratio of institutional investors, the higher environmental information disclosure quality. However, our conclusion may also have reverse causality, that is, the higher firm environmental information disclosure quality, the higher the shareholding proportion of institutional investors. This paper adopts the following two methods to alleviate this concern:(1) following the research of Boone and White (2015), we use Shanghai and Shenzhen 300 index (CSI 300 index) similar to the Russell 1,000/2,000 index as the instrumental variable. We believe that this instrumental variable meets the requirements of relevance and exogeneity: In terms of relevance, CSI 300 index will affect the shareholding ratio of institutional investors. At present, there is no evidence that CSI 300 index will affect environmental information disclosure quality, so it meets the exogenous principle. (2) We use one lag period shareholding ratio of institutional investors as instrumental variable; on this basis, we adopt the generalized method of moments (GMM) approach to control for a potential endogeneity bias in the original regression models.

The results of the endogenous tests are shown in Table 6. Among them, Columns (1) and (2) of Table 6 report the regression results of the impact of the shareholding ratio of institutional investors on environmental information disclosure quality when CSI 300 index is used as the instrumental variable. From the regression results in columns (1) and (2) of Table 6, we can find that there is still a significant positive correlation between the shareholding ratio of institutional investors and environmental information disclosure quality, and H1 is verified again.

**Table 6:** Institutional investors' heterogeneity and environmental information disclosure quality: endogenous test.

**Variable**	**(1)** ***Invh* OLS** **First stage regression**	**(2)** ***Eidq* OLS** **Second stage regression**	**(3)** ***Invh* GMM** **First stage regression**	**(4)** ***Eidq* GMM** **Second stage regression**
*Intercept*	−1.163*** (−21.27)	13.261*** (7.10)	−0.162*** (−6.07)	−4.336*** (−22.55)
*CSI_300_Index*	0.018** (2.47)			
*Invh_IV1*		14.648*** (9.68)		
*LagInvh*			0.877*** (168.20)	
*Invh_IV2*				0.206*** (5.06)
*Roa*	0.176*** (7.69)	−1.996*** (−7.14)	0.025** (2.41)	0.518*** (5.69)
*Growth*	0.002** (1.96)	−0.040** (−10.14)	0.006*** (4.09)	−0.018*** (−3.76)
*Lev*	0.001 (0.06)	−0.019 (−0.46)	−0.004 (−0.70)	−0.086* (−1.76)
*Size*	0.069*** (32.62)	−0.788*** (−7.26)	0.010*** (9.28)	0.237*** (31.22)
*Board*	0.075*** (6.58)	−0.641*** (−5.21)	0.008 (1.57)	0.476*** (9.97)
*Ddbl*	−0.080** (−2.04)	1.401*** (7.63)	−0.024 (−1.18)	0.382** (2.39)
*Dual*	−0.058*** (−13.67)	0.742*** (8.34)	−0.015*** (−7.30)	−0.102*** (−5.51)
*Year fixed effect*	Yes	Yes	Yes	Yes
*Industry fixed effect*	Yes	Yes	Yes	Yes
*Observations*	14,358	14,358	10,858	10,858
*R^2^*	0.224	0.212	0.863	0.197

t-statistics are in parentheses; *, **, and *** indicate significance at the 10, 5, and 1% level, respectively.

Columns (3) and (4) of Table 6 report the GMM estimate results of the impact of the shareholding ratio of institutional investors on environmental information disclosure quality when one lag period shareholding ratio of institutional investors is used as the instrumental variable. From the GMM estimate results in columns (3) and (4) of Table 6, we can find that there is still a significant positive correlation between the shareholding ratio of institutional investors and environmental information disclosure quality, and H1 is verified again.

### Alternative Measures of Institutional Investors' Heterogeneity and Environmental Information Disclosure Quality

To test the sensitivity of the research conclusion to the institutional investors' heterogeneity measurement, we follow the research of Elyasiani and Jia (2010), use the 5-year time window data to measure institutional investors' heterogeneity from the horizon dimension, and still use model (3) to test H2 in this paper.

To test the sensitivity of the research conclusion to the environmental information disclosure quality measurement, we also use the following two indicators as alternative environmental information disclosure quality measures: (1) divide the environmental information quality score by its possible maximum value of 50 as the measurement indicator of environmental information disclosure quality (*Eidq/Max)*. (2) The total score of environmental information quality is used as the measurement indicator of environmental information disclosure quality (*Eidq/Sum)*.

The results of the reconstructed regression are shown in Table 7. From column (1) of Table 7, we can find the newly defined measurement indicator of institutional investors' heterogeneity still significantly positively correlates with environmental information disclosure quality, which is in line with the expectation of H2. It shows that the research conclusion of this paper is not sensitive to the measurement indicator of institutional investors' heterogeneity. From columns (2) and (3) of Table 7, it can be found that the shareholding ratio of institutional investors is still significantly positively correlated with the two newly defined environmental information disclosure quality measurement indicators, which is in line with the expectation of H1. From columns (4) and (5) of Table 7, we can find that institutional investors' heterogeneity is significantly positively correlated with the two newly defined environmental information disclosure quality measurement indicators, which is in line with the expectation of H2; This shows that the research conclusion of this paper is not sensitive to the measurement indicators of environmental information disclosure quality.

**Table 7:** Institutional investors' heterogeneity and environmental information disclosure quality: measurement indicator sensitivity tests.

**Variable**	**(1)** ***Eidq***	**(2)** ***Eidq/Max***	**(3)** ***Eidq/Sum***	**(4)** ***Eidq/Max***	**(5)** ***Eidq/Sum***
*Intercept*	−4.954*** (−24.93)	−1.312*** (−37.29)	−65.625*** (−37.29)	−1.330*** (−38.54)	−66.502*** (−38.54)
*Invh*		0.031*** (5.29)	1.541*** (5.29)		
*Stable*	0.114*** (7.48)			0.020*** (7.91)	1.022*** (7.91)
*Roa*	0.818*** (6.72)	0.147*** (6.89)	7.344*** (6.89)	0.150*** (7.05)	7.495*** (7.05)
*Growth*	−0.139*** (−8.07)	−0.032*** (−10.62)	−1.611*** (−10.62)	−0.030*** (−9.76)	−1.488*** (−9.76)
*Lev*	−0.163*** (−3.48)	−0.026*** (−3.27)	−1.291*** (−3.27)	−0.028*** (−3.49)	−1.377*** (−3.49)
*Size*	0.265*** (35.26)	0.060*** (44.12)	2.998*** (44.12)	0.061*** (46.99)	3.053*** (46.99)
*Board*	0.472*** (10.26)	0.085*** (10.73)	4.256*** (10.73)	0.084*** (10.64)	4.213*** (10.64)
*Ddbl*	0.453*** (2.76)	0.052* (1.85)	2.616* (1.85)	0.051* (1.81)	2.562* (1.81)
*Dual*	−0.100*** (−5.53)	−0.016*** (−5.26)	−0.780*** (−5.26)	−0.016*** (−5.26)	−0.776*** (−5.26)
*Year fixed effect*	Yes	Yes	Yes	Yes	Yes
*Industry fixed effect*	Yes	Yes	Yes	Yes	Yes
*Observations*	12,137	15,937	15,937	15,937	15,937
*Adj. R^2^*	0.213	0.265	0.265	0.266	0.266

t-value is in brackets; * and *** indicate significance at the 10 and 1% level, respectively.

### Difference-GMM Dynamic Panel Analysis

To prove that environmental information disclosure quality and institutional ownership increase simultaneously rather than being common characteristics that tend to happen in the same firms, we use a difference-GMM dynamic panel model to carry out difference-GMM analysis on H1 and H2 in this paper. The difference-GMM results are shown in Table 8. There is no essential change in the research conclusion, and H1 and H2 are verified again.

**Table 8:** Institutional investors' heterogeneity and environmental information disclosure quality: difference-GMM dynamic panel analysis.

**Variable**	**(1)**	**(2)**
*Intercept*	9.998 (1.59)	3.439 (0.56)
*LagEdiq*	−0.032*** (−8.86)	−0.325*** (−8.81)
*Invh*	3.571*** (2.99)	
*Stable*		1.264*** (3.37)
*Roa*	41.392*** (9.27)	39.210*** (8.46)
*Growth*	−1.360* (−1.87)	−1.197 (−1.60)
*Lev*	3.405*** (2.70)	3.048** (2.36)
*Size*	−0.569** (−2.48)	−0.488** (−2.09)
*Board*	3.679* (1.79)	5.964*** (3.18)
*Ddbl*	−17.829*** (−3.19)	−15.518*** (−2.69)
*Dual*	−0.826 (−1.48)	−0.464 (−0.80)
*Firm fixed effect*	Yes	Yes
*Year fixed effect*	Yes	Yes
*Observations*	10,858	10,858
*Wald test*	0.000	0.000
*AR (2)*	−0.830	−0.24
*Sargan test*	1.000	1.000

z value is in brackets; *, **, and***are significant at 10, 5, and 1%, respectively.

### Full Sample Regression

The research conclusion of this paper may be caused by sample selection bias. To alleviate the impact of sample selection bias on the research conclusion of this paper, we use all listed firms of China's A-share as research samples to make regression analysis on H1 and H2 of this paper. The multiple regression results are shown in Table 9. There is no essential change in the research conclusion, and H1 and H2 are verified again.

**Table 9:** Institutional investors' heterogeneity and environmental information disclosure quality: full sample.

**Variable**	**(1)**	**(2)**
*Intercept*	−4.603*** (−32.37)	−4.781*** (−34.48)
*Invh*	0.179*** (6.87)	
*Stable*		0.080*** (7.07)
*Roa*	0.894*** (9.51)	0.927*** (9.91)
*Growth*	−0.142*** (−11.36)	−0.135*** (−10.74)
*Lev*	−0.099*** (−2.89)	−0.105*** (−3.04)
*Size*	0.250*** (43.89)	0.259*** (47.80)
*Board*	0.346*** (10.13)	0.350*** (10.26)
*Ddbl*	0.456*** (3.69)	0.444*** (3.59)
*Dual*	−0.106*** (−7.93)	−0.110*** (−8.29)
*Year fixed effect*	Yes	Yes
*Industry fixed effect*	Yes	Yes
*Observations*	20,140	20,140
*Adj. R^2^*	0.248	0.248

t-value is in brackets; ^***^ indicates significance at the 1% level.

## Additional Analysis

### Influence Mechanism Test

This paper believes that high-quality environmental information disclosure can reduce the degree of information asymmetry and alleviate agency conflict, so that institutional investors need high-quality environmental information disclosure to protect their own interests from infringement. Therefore, we use the following models (4) and (5) to test the impact of environmental information disclosure quality on the degree of information asymmetry and agency cost.


(4) $$\begin{array}{l} Opacity=\beta_{0}+\beta_{1}Eidq+\beta_{2}Roa+\beta_{3}Growth+\beta_{4}Lev\\ +\beta_{5}Size+\beta_{6}Board\\ +\beta_{7}Ddbl+\beta_{8}Dual+\sum Year+\sum Industry+\varepsilon \end{array}$$

(5) $$\begin{array}{l} Agc=\beta_{0}+\beta_{1}Eidq+\beta_{2}Roa+\beta_{3}Growth+\beta_{4}Lev+\beta_{5}Size\\ +\beta_{6}Board\\ +\beta_{7}Ddbl+\beta_{8}Dual+\sum Year+\sum Industry+\varepsilon \end{array}$$

In model (4), *Opacity* represents the degree of information asymmetry. We follow the idea of Hutton et al. (2009), use the modified Jones model to estimate the firm's discretionary accruals by year and industry, and take the mean of absolute values for 3 consecutive years to measure the degree of information asymmetry. The larger the value of this index is, the higher the degree of information asymmetry. The definition of other variables is the same as that of model (2).

In model (5), *Agc* represents the agency cost, which is measured by dividing the sum of administrative expenses and sales expenses by operating revenue. The definition of other variables is the same as that of model (2).

Columns (1) and (2) of Table 10 report the multiple regression results of environmental information disclosure quality on the degree of information asymmetry and agency cost, respectively. From columns (1) and (2) of Table 10, it can be found that environmental information disclosure quality is significantly negatively correlated with the degree of information asymmetry and agency cost, respectively, which further verifies the theoretical premise of this research hypothesis.

**Table 10:** Influence mechanism test.

**Variable**	**(1) *Opacity***	**(2)** ***Agc***
*Intercept*	0.088*** (12.66)	0.218*** (7.71)
*Eidq*	−0.002*** (−7.24)	−0.010*** (−7.98)
*Roa*	−0.028*** (−6.79)	−0.251*** (−14.47)
*Growth*	0.007*** (10.16)	−0.019*** (−6.93)
*Lev*	0.021*** (13.40)	−0.156*** (−24.38)
*Size*	−0.003*** (−9.81)	−0.003*** (−2.59)
*Board*	−0.002 (−1.36)	0.015** (2.26)
*Ddbl*	0.016*** (2.94)	0.071*** (3.15)
*Dual*	0.002*** (2.64)	0.011*** (4.57)
*Year fixed effect*	Yes	Yes
*Industry fixed effect*	Yes	Yes
*Observations*	13,175	15,588
*Adj. R^2^*	0.069	0.109

t-statistics are in parentheses; ** and *** indicate significance at the 5 and 1% level, respectively.

### Group Test of Institutional Investors' Heterogeneity

Based on the research of Elyasiani and Jia (2010), research sample of this paper is divided into groups according to institutional investors' heterogeneity to further the impact of institutional investors' shareholding on the environmental information disclosure quality in the unstable and stable groups. According to the definitions of unstable and stable institutional investors, we believe that the positive correlation between the shareholding ratio of institutional investors and environmental information disclosure quality is more significant in the stable group than in the unstable group.

Columns (1) and (2) of Table 11, respectively, report the impact of the shareholding ratio of institutional investors in unstable and stable groups on environmental information disclosure quality. From columns (1) and (2) of Table 11, it can be found that the positive correlation between the shareholding ratio of institutional investors and environmental information disclosure quality is more significant in the stable group than in the unstable group.

**Table 11:** Shareholding ratio of institutional investors and environmental information disclosure quality: unstable group and stable group test.

**Variable**	**(1)** ***Stable*=0**	**(2)** ***Stable*=1**
*Intercept*	−4.690*** (−15.58)	−4.664*** (−19.30)
*Invh*	0.002 (0.05)	0.121** (2.20)
*Roa*	1.317*** (8.13)	0.514*** (3.09)
*Growth*	−0.155*** (−7.37)	−0.170*** (−5.83)
*Lev*	−0.119* (−1.88)	−0.081 (−1.36)
*Size*	0.262*** (22.61)	0.258*** (27.10)
*Board*	0.412*** (6.42)	0.457*** (7.96)
*Ddbl*	0.344 (1.52)	0.235 (1.14)
*Dual*	−0.104*** (−4.78)	−0.073*** (−3.10)
*Year fixed effect*	Yes	Yes
*Industry fixed effect*	Yes	Yes
*Observations*	6,999	7,359
*Adj. R^2^*	0.166	0.227

t-statistics are in parentheses; *, **, and *** indicate significance at the 10, 5, and 1% level, respectively.

### Group Test of Environmental Information Disclosure Regulatory Policies

Chinese government has decided to regulate environmental information disclosure since 2003. The announcement on firm environmental information disclosure issued by the former State Environmental Protection Administration in 2003 can be said to be the first regulation on firm environmental information disclosure in China. In 2008, the measures for environmental information disclosure (trial implementation) issued by the former State Environmental Protection Administration came into force. In 2015, the measures were replaced by the measures for environmental information disclosure of firms and institutions. At the end of 2021, Ministry of Ecology and Environment of the people's Republic of China promulgate the measures for the administration of firm environmental information disclosure, which officially came into force on 8 February 2022. The newly promulgated measures for the administration of firm environmental information disclosure unify the previously scattered provisions on the legal disclosure of firm environmental information and focus on solving the main crux affecting the effective promotion of firm information disclosure, which has a more clear and practical guiding significance for disclosure firm.

The sample period of this paper is from 2008 to 2020, which provides an opportunity for us to test whether the measures for environmental information disclosure of firms and institutions promulgated in 2015 have significantly improved environmental information disclosure quality. Taking 2015 as the regulatory policy time node, we test whether there is a significant difference in the mean of environmental information disclosure quality before and after 2015. The mean test results of independent samples are shown in Table 12. It can be seen from Table 12 that after the promulgation of the measures for environmental information disclosure of firms and institutions in 2015, environmental information disclosure quality has been significantly improved than before, which shows that China's environmental information disclosure regulatory policy has achieved practical results.

**Table 12:** Univariate test.

**Variable**	***Eidq Mean***
*Firms_before*2015(1)	10.03
*Firms_after*2015(2)	11.73
(2)-(1)	1.70
*Difference-in-differences test*	10.96***

***Indicate significance at the 1% level.

### Group Test of Property Right Nature

We believe that different property rights may affect the shareholding of institutional investors, and the conclusion of this paper may be due to property rights nature. Therefore, according to firm property rights nature, we divide the samples into state-owned firms and non-state-owned firms (*Soe*, the value of state-owned enterprises is 1, otherwise it is 0). We regress H1 in the samples of state-owned firms and non-state-owned firms, respectively. The regression results are shown in Table 13. Within two subsamples, the coefficient of *Invh* is not significant, which indicates that property rights nature does not affect the results in Table 5.

**Table 13:** Shareholding ratio of institutional investors and environmental information disclosure quality: group test of state-owned and non-state-owned firms.

**Variable**	**(1) *Soe*=1**	**(2)** ***Soe*=0**
*Intercept*	−4.484*** (−16.87)	−4.150*** (−13.80)
*Invh*	0.104 (1.62)	0.025 (0.66)
*Roa*	−0.072 (−0.34)	1.511*** (10.44)
*Growth*	−0.117*** (−4.10)	−0.181*** (−8.41)
*Lev*	−0.438*** (−6.35)	0.030 (0.51)
*Size*	0.266*** (24.86)	0.237*** (21.88)
*Board*	0.361*** (5.78)	0.400*** (6.46)
*Ddbl*	0.067 (0.30)	0.368* (1.70)
*Dual*	0.082** (2.27)	−0.089*** (−4.76)
*Year fixed effect*	Yes	Yes
*Industry fixed effect*	Yes	Yes
*Observations*	5,442	8,442
*Adj. R^2^*	0.214	0.156

t-statistics are in parentheses; *, **, and *** indicate significance at the 10, 5, and 1% level, respectively.

## Conclusions

Based on the dividing institutional investors into the stable and the unstable institutional investors, this paper uses the data of listed firms in China's A-share heavy pollution industry between 2008 and 2020 and explores the effect of institutional investors' heterogeneity on environmental information disclosure behavior from the perspective of environmental information disclosure quality. Empirical evidence shows that the higher the shareholding ratio of institutional investors, the higher environmental information disclosure quality, which verifies the hypothesis of effective monitor of institutional investors on firm environmental information disclosure behavior. Further analysis shows that compared with the unstable institutional investors, the stable institutional investors have significant positive impact on environmental information disclosure quality, which shows that stable institutional investors can effectively monitor the firm's environmental information disclosure behavior, strengthen the main responsibility of firm ecological and environmental protection, and promote the green development of firms. After a series of robustness tests, the research conclusion is still valid. It can be seen that institutional investors are an important factor that affects environmental information disclosure quality, and there are significant differences in the impact of different types of institutional investors on environmental information disclosure quality.

Conclusions of this paper have important theoretical and practical significance. First, this paper finds that there are significant differences in the impact of different types of institutional investors on environmental information disclosure quality, which breaks through the previous research on the impact of institutional investors on firm behavior and decision-making only from the overall perspective of institutional investors. Second, the research conclusion of this paper shows that we should comprehensively and objectively understand the impact of institutional investors on environmental information disclosure quality, especially compared with the unstable institutional investors, the stable institutional investors play an effective monitor role and can significantly improve environmental information disclosure quality. It requires the regulators to further improve the policy of vigorously developing the team of institutional investors, guide institutional investors to pay attention to the long-term investment in the firm, timely monitor the firm's environmental information disclosure behavior, and promote the firm to fully disclose high-quality environmental information to external investors.

The limitations of this paper are as follows: First, the problem of omitting time variable characteristics. Although this paper adopts Shanghai and Shenzhen 300 index (CSI 300 index) similar to the Russell 1,000/2,000 index as the instrumental variable, one lag period shareholding ratio of institutional investors as instrumental variable was used, the generalized method of moments (GMM) approach was adopted to control for a potential endogeneity bias in the original regression models, and difference-GMM dynamic panel model was utilized to carry out the difference-GMM analysis. But this paper still has the problem of omitting variable. For example, foreign ownership or competition or exports may be increasing and this may simultaneously stimulate firms to disclose more environmental information and also attract institutional investors. Second, we regard institutional investors as a whole and investigate their impact on environmental information disclosure quality, the impact of different types of institutional investors such as investment funds, pension funds, financial companies, and QFII on environmental information disclosure quality is not considered.

## Data Availability Statement

The original contributions presented in the study are included in the article/supplementary material, further inquiries can be directed to the corresponding author.

## Author Contributions

ZL contributed to conception, data analysis, and writing. TZ and XZ contributed to conception and writing. YZ contributed to writing and checking. All authors contributed to this article and agreed to the submitted version.

## Funding

This paper was supported by the National Social Science Foundation of China (No. 20BJY030).

## Conflict of Interest

The authors declare that the research was conducted in the absence of any commercial or financial relationships that could be construed as a potential conflict of interest.

## Publisher's Note

All claims expressed in this article are solely those of the authors and do not necessarily represent those of their affiliated organizations, or those of the publisher, the editors and the reviewers. Any product that may be evaluated in this article, or claim that may be made by its manufacturer, is not guaranteed or endorsed by the publisher.
